# Conquering the cytokine storm in COVID‐19‐induced ARDS using placenta‐derived decidua stromal cells

**DOI:** 10.1111/jcmm.16986

**Published:** 2021-10-10

**Authors:** Behnam Sadeghi, Elham Roshandel, Ali Pirsalehi, Sepide Kazemi, Ghazaleh Sankanian, Mohammad Majidi, Maryam Salimi, Nasser Aghdami, Hoda Sadrosadat, Sarvenaz Samadi Kochaksaraei, Farshid Alaeddini, Olle Ringden, Abbas Hajifathali

**Affiliations:** ^1^ Translational Cell Therapy Research (TCR) Department of Clinical Science, Intervention and Technology CLINTEC Karolinska Institutet Huddinge Sweden; ^2^ Hematopoietic Stem Cell Research Center Shahid Beheshti University of Medical Sciences Tehran Iran; ^3^ Department of Internal Medicine School of Medicine Taleghani Hospital Shahid Beheshti University of Medical Sciences Tehran Iran; ^4^ Advanced Therapy Medicinal Product (ATMP) Breast Cancer Research Center Motamed Cancer Institute ACECR Tehran Iran; ^5^ Department of Tissue Engineering & Regenerative Medicine Faculty of Advanced Technologies in Medicine Iran University of Medical Sciences Tehran Iran; ^6^ Department of Regenerative Medicine, Cell Science Research Center Royan Institute for Stem Cell Biology and Technology, ACECR Tehran Iran; ^7^ Department of Infectious Diseases and Tropical Medicines Tehran University of Medical Sciences Tehran Iran; ^8^ Research Center for Health Management in Mass Gathering Red Crescent Society of the Islamic Republic of Iran Tehran Iran

**Keywords:** COVID‐19, cytokine storm, decidua stromal cells, intensive care unit, survival

## Abstract

Acute respiratory distress syndrome (ARDS) is the most common cause of death in COVID‐19 patients. The cytokine storm is the main driver of the severity and magnitude of ARDS. Placenta‐derived decidua stromal cells (DSCs) have a stronger immunosuppressive effect than other sources of mesenchymal stromal cells. Safety and efficacy study included 10 patients with a median age of 50 (range 14–68) years with COVID‐19‐induced ARDS. DSCs were administered 1–2 times at a dose of 1 × 10^6^/kg. End points were safety and efficacy by survival, oxygenation and effects on levels of cytokines. Oxygenation levels increased from a median of 80.5% (range 69–88) to 95% (range 78–99) (*p* = 0.012), and pulmonary infiltrates disappeared in all patients. Levels of IL‐6 decreased from a median of 69.3 (range 35.0–253.4) to 11 (range 4.0–38.3) pg/ml (*p* = 0.018), and CRP decreased from 69 (range 5–169) to 6 (range 2–31) mg/ml (*p* = 0.028). Two patients died, one of a myocardial infarction and the other of multiple organ failure, diagnosed before the DSC therapy. The other patients recovered and left the intensive care unit (ICU) within a median of 6 (range 3–12) days. DSC therapy is safe and capable of improving oxygenation, decreasing inflammatory cytokine level and clearing pulmonary infiltrates in patients with COVID‐19.

## 1 INTRODUCTION

The COVID‐19 pandemic has led to the death of more than 4.2 million people worldwide, with more than 196 million infections reported.^1^ The COVID‐19 infection may be asymptomatic or cause mild symptoms like fever, cough, fatigue and muscle soreness.^2^ Some patients suddenly deteriorate and develop acute respiratory distress syndrome (ARDS) and multiple organ failure (MOF), which may lead to death. Among patients who require hospitalization for COVID‐19 infection, mortality rates range from 5% to 15%.^3^, ^4^

Acute respiratory distress syndrome and acute lung injury (ALI) are characterized by rapid alveolar injury, inflammation, neutrophil accumulation, pulmonary oedema and impaired carbon dioxide elimination.^5^ Abnormal and uncontrolled production of cytokines was observed in critically ill patients with COVID‐19 pneumonia.^6^ One of the most dramatic features of the pathophysiology of ARDS in COVID‐19 is the cytokine storm with elevated levels of several acute inflammatory mediators, *for example* IL‐6, C‐reactive protein (CRP) and lactate dehydrogenase (LDH).^7^

There is no specific therapy for COVID‐19 or specifically for ARDS/ALI, and most investigated regimens have little or no effect on hospitalized patients with COVID‐19.^8^ Treatment is generally symptomatic, including oxygen supply and ventilatory support to maintain good oxygenation and adequate carbon dioxide elimination. Most deaths from COVID‐19 (88%) are due to ARDS.^9^ Blocking the cytokine storm and consequently preventing ARDS is a main factor in reducing COVID‐19‐related mortality.^7^ Mesenchymal stromal cells (MSC) constitute a known cell‐based therapy for acute inflammatory diseases, and MSCs have been applied in several clinical trials.^10^, ^11^, ^12^ Among all stromal cells, placenta‐derived decidua stromal cells (DSCs) have been shown to have a strong immunomodulatory effect in clinical studies and in vitro assays.^13^

Compared to bone marrow‐derived (BM)‐MSC, DSCs have significantly stronger immunomodulatory and anti‐inflammatory properties.^14^ DSCs can successfully treat and control steroid‐refractory acute graft‐versus‐host disease (aGVHD).^13^ Intravenous (i.v.) infusion of DSCs was safe both in human and animals.^15^, ^16^ Clinical and experimental data support the use of DSCs or MSCs for septicaemia and ARDS.^17^, ^18^ However, two randomized clinical trials using adipose and BM‐MSCs, respectively, failed to show efficacy.^17^, ^19^

We hypothesized that DSCs may reduce ARDS‐induced mortality in COVID‐19 patients. Here, we report the use of DSCs for the treatment of COVID‐19‐induced ARDS/ALI in a phase 1/2 trial.

## 2 MATERIALS AND METHODS

### 2.1 Patients

From March to November 2020, ten COVID‐19 ARDS patients were treated with DSCs (Table 1). COVID‐19 infection was confirmed by quantitative reverse‐transcriptase polymerase chain reaction (qRT‐ PCR). This was a case series study, and recruitment was based on inclusion and exclusion criteria (Table 2).

**TABLE 1:** Patients characteristic

UPN	Age	Sex	Co‐morbidity	Days to admission	Antiviral treatment	Corticosteroid	Length of stay in ICU (days)	Confirmed COVID‐19 RT‐PCR	Outcome
9901	68	Male	IHD	7	Yes	Yes	9	−	Death
9902	34	Male	—	6	Yes	Yes	3	+	Recovered
9903	14	Male	—	3	Yes	No	—	+	Recovered
9904	56	Male	Epilepsy	14	No	No	9	+	Death
9905	51	Male	DM	14	No	No	6	−	Recovered
9906	50	Male	DM, HTN	7	Yes	Yes	11	+	Recovered
9907	38	Male	—	3	Yes	Yes	12	+	Recovered
9908	44	Female	HTN	10	Yes	Yes	6	−	Recovered
9909	50	Male	DM, Hyperlipoproteinaemia	3	Yes	Yes	8	−	Recovered/Death^a^
9910	63	Female	Breast cancer	7	Yes	Yes	6	−	Recovered

Abbreviations: DM, diabetes mellitus; DSC, decidua stromal cells; HTN, hypertension; IHD, ischaemic heart disease; UPN, unique patient number.

^a^ Despite achieving a partial response to the DSC therapy, this patient stopped taking medications in the middle of the treatment course on his own initiative. The patient left the hospital against recommendations by the physicians and staff and accepted the responsibility and risk on his own.

**TABLE 2:** Inclusion and exclusion criteria in the study

Inclusion criteria	1. Patients age between 12–75 years old
	2. Informed consent form
	3. Confirmed infection with SARS‐CoV‐2 using qRT‐ PCR or
	4. Clinical manifestation of COVID‐19 infection with lung involvement
	5. Pneumonia confirmed by CT scan or chest radiography
	6. Oxygen saturation ≤90%
	7. Respiration Rate (RR) ≥30 breath/min
	8. Difficulty breathing, Dyspnoea
Exclusion criteria	1. Patients with malignancies/severe systemic diseases/psychosis
	2. Patients with serious underlying diseases that affect survival including blood diseases, cachexia, active bleeding and severe malnutrition
	3. Patients with obstructive pulmonary pneumonia, severe interstitial fibrosis, alveolar proteinosis, allergic alveolitis and other known viral or bacterial pneumonias
	4. Continued use of immunosuppressive drugs or organ transplantation in the last 6 months
	5. In vitro life support (ECMO, ECCO2R, RRT)
	6. Predict death within 24 h
	7. Allergy to albumin or any components that be used for preparation of cell products
	8. Patients participating in other clinical trials
	9. Inability to provide informed consent or comply with study conditions

Abbreviation: CT, computed tomography.

The ethical committee of the Motamed Cancer Institute, Tehran, Iran, approved the isolation of DSCs and the application of DSCs for the treatment of ARDS patients (No. IR.ACECR.IBCRC.REC.1395.13). The study was also registered in the Iranian Registry of Clinical Trials (IRCT2017010531786N1). Informed consent was obtained from all patients and for the 14 years old boy, signed by his mother. The patients had full access to all available COVID‐19 medications.

The patients were evaluated by the treating physicians.

### 2.2 Decidua stromal cells isolation and expansion

Isolation, expansion and DSC preparation for injection have been explained elsewhere; briefly, the foetal membranes were dissected from the placenta tissues and underwent enzymatic digestion.^13^, ^14^, ^20^ DSCs were isolated and expanded under good manufacturing practice (GMP) condition from three donors, after caesarean section. Isolated DSCs were characterized by flow cytometry, evaluated for karyotype analysis and origin (maternal) and expanded until passage five (P5). Immunosuppressive capacity of DSC from passage 2 to passage 5 is similar in clinical trial and in vitro assays.^13^, ^21^ Subsequently, DSCs were frozen slowly in foetal bovine serum (FBS, GIBCO, Germany) containing 5% dimethyl sulphoxide (DMSO, Sigma, Germany) and stored in liquid nitrogen until use. All materials were GMP grade or GMP compliant qualification, and the FBS was previously used in clinical trials for acute GVHD and haemorrhages. Upon request from ICU units and based on patient weight (1 × 10^6^ DSCs/kg body weight), the required number of vials was thawed rapidly in an incubator/water bath (37°C), and the cell suspension was resuspended in infusion buffer, which was prepared from sodium chloride 0.9% (saline) buffer supplemented with 5% human serum albumin, (CSL; Behring). Cell numbers and viability were calculated, and if the cell viability was less than 90%, the batch was not qualified for infusion. Optimal dose of DSCs (1 × 10^6^ DSCs/kg body weight) and safety were previously evaluated for acute GVHD and haemorrhagic cystitis.^13^, ^15^, ^22^ DSCs were resuspended in an appropriate volume of infusion buffer to achieve a concentration of 2 × 10^6^ DSCs/ml. The cell suspension was transferred into a heparinized syringe (V‐med) and shipped to the hospital at room temperature in a biologic sample box within 30 min.

### 2.3 Cell infusion procedure

To prevent allergic reactions, 10 mg chlorphenamine (10 mg/ml) or 100 mg hydrocortisone was administered intravenously 10 min before each cell infusion. All DSC infusions were administered via a peripheral intravenous catheter (20‐gauge, pink, bore 1 mm). Before and after each DSC infusion, the venous line was flushed with 5 ml low molecular weight heparin (Caspian Tamin) at a dose of 1000 IU/ml. For patients needing more than one dose, DSCs were infused after 3 days.

### 2.4 Cytokine measurements

Before and after the DSC infusions, blood samples were collected from the patients. The blood was immediately centrifuged, and the serum was separated, labelled and immediately frozen and stored at −80°C until analysis. ELISA was carried out to determine the serum levels of cytokines including CCL2, IL‐6 and GCSF using R&D kits (R&D Systems) according to the manufacturer's instructions. The absorbance of each well was determined using a microplate spectrophotometer (epoch 2; BioTek) at 450 nm, and the cytokine concentrations were estimated by referring to the standard curve. Due to excessive workload, it was not possible to measure cytokines for all patients.

### 2.5 Flow cytometry

To explore the DSC phenotype, cells were stained with the following antibodies according to the manufacturer's instructions: CD14, CD45, CD90, CD73, CD29, CD105 and CD11b (all from BD Bioscience). A fluorescence‐activated cell sorter (FACS) machine, BD FACS Calibur (BD Biosciences), was used. CD142 was not determined on the DCSs used in this study.

### 2.6 CT scan

Computed tomography (CT) scans were performed using a commercial CT scanning system, SOMATOM Emotion eco (16‐slice configuration) (SIEMENS). For intubated or unconscious patients, chest X‐rays were performed.

### 2.7 Statistical analysis

Results are shown as individual and cumulative data, as median values. For analysis before and after DSC treatment, Wilcoxon signed rank tests were used for related samples. Pre‐intervention data are related to the day of infusion (before the DSC infusion), and post‐intervention data are related to the day after the last DSC infusion. All statistical analyses were performed with the SPSS software version 22. *p*‐Values of less than 0.05 were considered statistically significant.

## 3 RESULTS

### 3.1 DSC characterization

**FIGURE 1:**
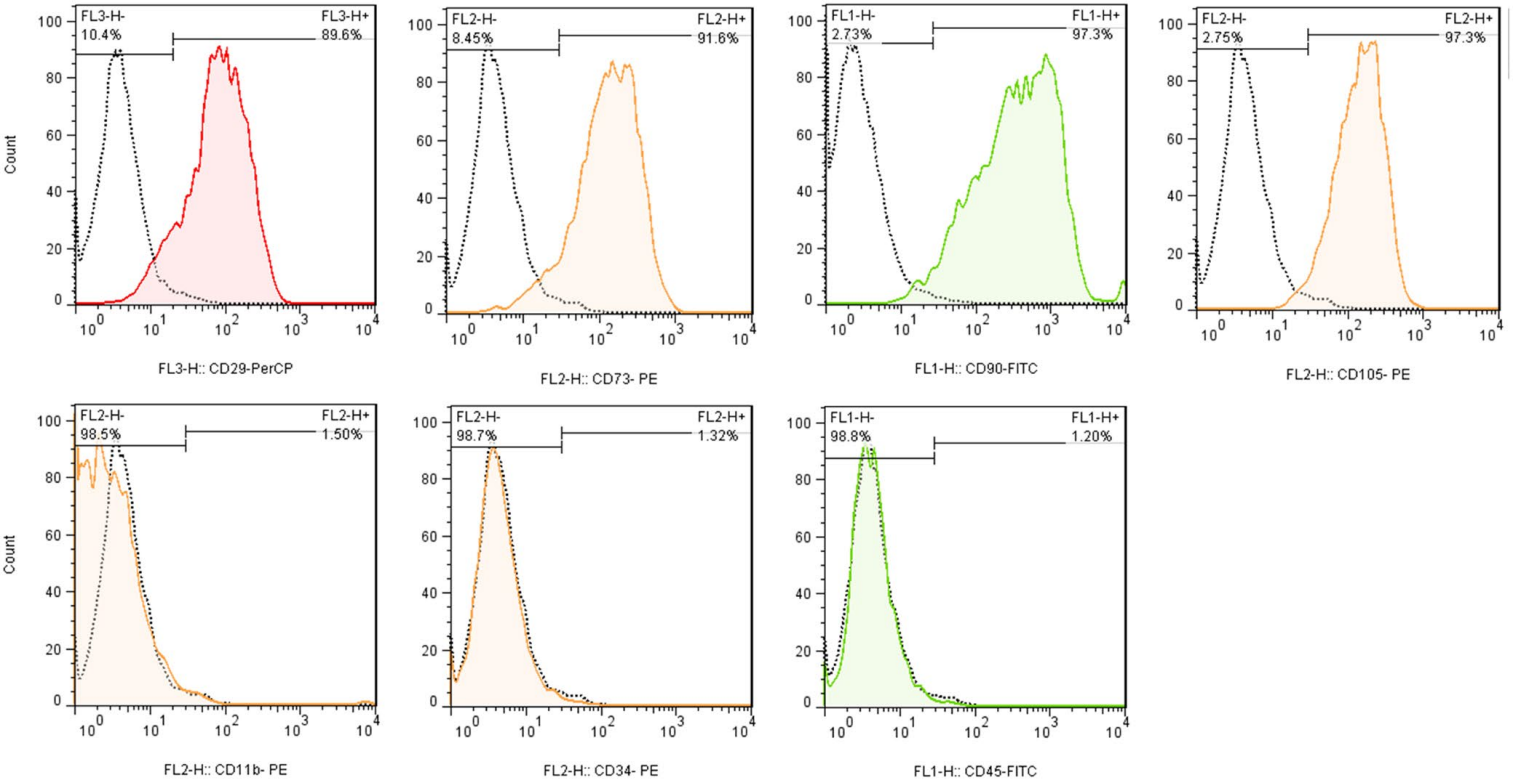
Phenotypic analysis of decidua stromal cells (DSCs) by flow cytometry. The coloured graphs show the expression of the different cell surface markers (filled areas under the curve) compared to unstained DSC controls (white areas under the curve). The percentages represent the size of each population as a proportion of the whole population

Decidua stromal cells were positive for CD29, CD73, CD90 and CD105, but negative for CD11b, CD34 and CD45 (Figure 1). DSCs were maternal and showed normal karyotype.

### 3.2 Infusion information and DSC data

Patients received 2 infusions (range 1–3). Median DSC dose was 1.02 x106 cells/kg (range 0.85–1.23). DSCs were at passage 4 (P4) and/or P5 (only one DSC infusion was at P3). DSC viability at infusion was median 94% (range 90%–96%).

### 3.3 Patient outcomes

#### 3.3.1 Patient 1 (UPN 9901)

A 68‐year‐old man with ischaemic heart disease (IHD) had cough, fever and shivering. The patient was alert (Glasgow Coma Scale, GCS 15/15), but fatigued. CT scan showed ground‐glass opacities distributed to the periphery >50% of lung tissue. Peripheral oxygen saturation (SpO_2_) was 88% at admission, and the patient was given 8 L/min oxygen via face mask. Oxygen consumption increased (10 L/min), but oxygen saturation did not improve (88%) despite bilevel positive airway pressure (BiPAP). Next day, oxygen saturation dropped to 60%; he was intubated, connected to a ventilator and GCS dropped to 10/15. Because of deterioration, DSC therapy was considered the only option. Five days after admission, DSCs were infused (1 × 10^6^ /kg). Immediately, SpO_2_ increased from 71% to 88%. The oxygen saturation remained unstable but never below 88%. DSCs were reinfused 2 days after the first dose, but the patient did not improve; after 24 h, the patient died due to cardiac arrest and multiorgan failure. Following DSC infusions, IL‐6, G‐CSF and CRP declined (Table 3).

**TABLE 3:** Cytokine and oxygen saturation levels in each patient before and after DSC therapy

UPN	CCL2		GCSF		IL‐6		CRP		O_2_ saturation without mask	
	Base^a^	After^b^	Base	After	Base	After	Base	After	Base	After
9901	585.8	805	155	37.8	253.4	30.7	169	29	60	—
9902	214.4	47.5	75.5	56.8	69.3	4.27	8	6	85	96
9903	396.5	297.9	54.9	89.5	41	18.8	45	21	88	97
9904	335.3	106.1	197.3	142.9	115.5	38.3	142	31	—	—
9905	695.3	NA	84.3	NA	78.3	NA	69	NA	88	99
9906	254.5	197.3	142.4	44.2	35	5.6	97	5	78	94
9907	217.5	232.6	31.6	84.1	62.6	4	102	6	81	98
9908	—	—	—	—	—	—	7.1	NA	80	86
9909	—	—	—	—	—	—	28.9	NA	77	78
9910	—	—	—	—	—	—	9.8	NA	69	85
Median	335.3	215.0	84.3	70.5	69.3	11.0	57.0	13.5	80.0	95.0
PV	0.345		0.249		0.028		0.028		0.012	

Abbreviations: DSC, decidua stromal cells; UPN, unique patient number.

^a^ Base = 1 day before DSC therapy.

^b^ After = 1 day after the last DSC infusion or on the day of discharge.

#### 3.3.2 Patient 2 (UPN 9902)

**FIGURE 2:**
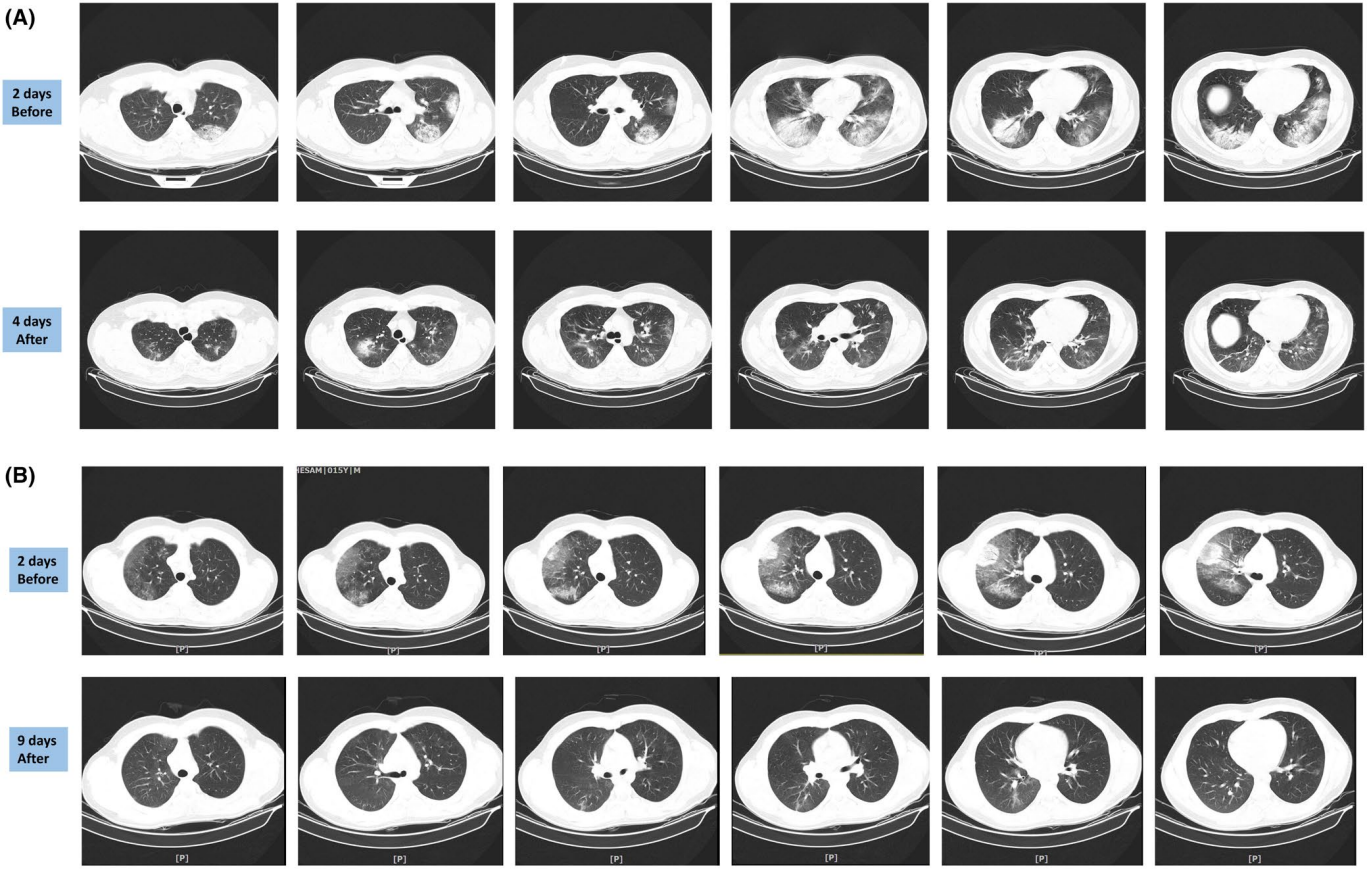
Computed tomography (CT) scan images of the lungs in patients with COVID‐19 infection before and after decidua stromal cells (DSC) therapy. (A) Lung CT scan of patient 9902, 2 days before and 4 days after DSCs treatment (1 day after the second DSC dose). For more information, refer to the text. (B) Lung CT scan of patient 9903, 2 days before and 9 days after DSC treatment (4 days after the second DSC dose). For more information, refer to the text. For a better overview of the changes, live CT scan images are available as Supplementary Files

A 34‐year‐old man was admitted with fever, shivering, night‐time sweating, nausea, vomiting, dyspnoea and oxygen saturation of 86%. Oxygen was initiated (7 L/min) via face mask. CT scan showed ground‐glass opacities >50% of both lungs, mainly middle lobe. The patient developed severe dyspnoea. DSCs were infused 2 days after admission, and the second dose was given 3 days later. Levels of IL‐6, G‐CSF, CRP and CCL2 decreased dramatically (Table 3). Twenty four hours after the first dose of DSCs, the patient felt less chest tightness, breathing improved, and the frequency and intensity of coughing decreased to an extent that the patient was able to speak. Six days after the second DSC dose, the patient was discharged from the hospital with oxygen saturation >96% (Table 3). The opacities and infiltration in the middle and bottom of both lungs disappeared 4 days after the first DSC dose (Figure 2A).

#### 3.3.3 Patient 3 (UPN 9903)

A 14‐year‐old boy was admitted with 3 days of fever, cough, severe myalgia, abdominal pain, dyspnoea and a sore throat. Lymphadenopathies were observed at the neck and axillaries. Oxygen saturation was 94% with 7 L/min oxygen via face mask. CT scan showed unilateral ground‐glass opacity from the centre to the periphery (Figure 2B). CT scan revealed hepatomegaly, splenomegaly and significant mesenteric lymphadenopathy. Despite the lung involvement, oxygen saturation levels remained above 94% with an oxygen supply of 6 L/min. Lung infiltration increased; the patient felt increasingly weak and fatigued, and oxygen saturation dropped to 88% within 24 h. DSC therapy was therefore considered. The patient received two doses of DSCs, 3 and 7 days after hospitalization. After the second DSC dose, the patient could walk and showed improvements. CT scan showed clearance of infiltration and opacities (Figure 2B). Levels of IL‐6, CRP and CCL2 decreased, and oxygen saturation levels recovered to >97% (Table 3). He was discharged 3 days after the second DSC dose.

#### 3.3.4 Patients 4 (UPN 9904)

A 56‐year‐old man was admitted with a medical history of epilepsy/convulsion and chronic alcohol consumption for 3 years. At admission, the patient had discontinued his anti‐epileptic medications on his own initiative. He had angina pectoris with ST elevation, dyspnoea, respiratory distress and decreased consciousness. Dry cough and chest pain had appeared 2 weeks before. Oxygen 10 L/min was administered via face mask, but dyspnoea worsened, and he developed a myocardial infarction during CT scan imaging. The patient lost consciousness, developed convulsions and was intubated. On the ventilator, the patient's oxygen saturation was 95%. The patient experienced convulsions and was treated with anticonvulsive medication as well as therapy for the myocardial infarction. He became worse, and convulsions could not be controlled. Two days after admission to the ICU, he received two doses of DSC therapy 3 days apart. The patient died 9 days after admission from multiorgan failure. However, IL‐6, G‐CSF, CRP and CCL‐2 decreased after two doses of DSC therapy (Table 3).

#### 3.3.5 Patients 5 (UPN 9905)

A 51‐year‐old man, with diabetes mellitus and congestive heart failure (CHF), was admitted with fever, dyspnoea, fatigue, anorexia and diarrhoea. Oxygen saturation was 77%, and oxygen 6 L/min was administered via face mask. CT scan showed >50% focal ground‐glass lung‐infiltrations. Dyspnoea became worse, and the oxygen supply was increased to 10 L/min via face mask. Due to worsening, the patient was treated with a reservoir bag supplying 10 L/min, while the blood oxygen level was 88%. The patient had uncontrolled diabetes mellitus, prohibiting corticosteroids. DSCs were infused 3 days after hospitalization. 24 h after DSC infusion, blood level of oxygen reached 90% with the oxygen supply decreased to 4 L/min. Cytokine levels normalized (Table 3). Two days after DSC infusion, dyspnoea disappeared, and the patient was transferred from the ICU to a medical ward. Twenty four hours later, oxygen saturation exceeded 99% without oxygen supply, and the patient was discharged from the hospital.

#### 3.3.6 Patients 6 (UPN 9906)

A 49‐year‐old man was admitted with a medical history of diabetes mellitus and hypertension for more than 10 years. A week prior to admission, the patient had developed fever, shivering, abdominal pain, diarrhoea, myalgia, cough and dyspnoea. Lung CT scan showed diffuse bilateral ground‐glass. Oxygen saturation on admission was 74% (without a mask), and an oxygen supply of 10 L/min was applied using a face mask. Despite these medications and supportive care, oxygen saturation levels did not improve. The face mask was replaced by a reservoir bag supplying 15–20 L/min. Oxygen saturation levels barely reached 78% 3 days after admission. Therefore, DSC infusion was administered 3 days after admission. Oxygen saturation levels stabilized. A second DSC dose was infused 3 days later, and oxygen saturation levels increased to 92%. Dyspnoea and cough improved, and a recovery process started. Plasma cytokine levels decreased following DSC infusions (Table 3). Due to improvements, the physicians decided to infuse a third dose of DSCs, 9 days after admission. Two days later, the patient was discharged with normal oxygen saturation (>95%) and no dyspnoea.

#### 3.3.7 Patients 7 (UPN 9907)

A 38‐year‐old man was admitted with dyspnoea, fever and cough. Oxygen saturation was 78% (without a face mask); therefore, an oxygen supply of 8 L/min via face mask was commenced. Three days after, admission oxygen saturation was 81% (without a mask). Therefore, oxygen supply shifted to reserve bag. The patient was dyspnoeic with high levels of IL‐6. Three days after admission, with no improvement in oxygenation and a lung CT scan showing infiltration, the patient was considered for DSC therapy.

The first dose of DSCs was injected 3 days after admission. A second dose was given 3 days later, and oxygen saturation levels reached 93%. After that, the patient maintained an oxygen level >98% (Table 3) without a face mask. IL‐6 levels decreased after the DSC therapy (Table 3). Six days after the cell therapy, the patient was discharged in good health.

#### 3.3.8 Patients 8 (UPN 9908)

A 44‐year‐old woman with a history of hypertension was admitted with myalgia, fever, shivering, dry cough and dyspnoea. Chest CT scan showed multifocal bilateral ground‐glass infiltration of the lungs. Due to dyspnoea and low oxygen saturation (82% without a mask), oxygen 10 L/min was administered using a reservoir bag, but blood oxygen saturation levels remained at 85%. Lung involvement worsened, and oxygen saturation decreased to 80%. The reservoir bag was switched to BiPAP. With no improvement, DSCs were infused 5 days after admission. One day after the DSC infusion, the patient stabilized, and 2 days after DSC infusion, oxygen saturation reached 95% (without a mask). Therefore, the BiPAP was switched to a reservoir bag (10 L/min). The dyspnoea improved, and cough intensity decreased. After that, the patient was transferred from the ICU to a regular ward. Oxygen supply was switched to a nasal tube (7 L/min). The patient was discharged in good health, 19 days after admission to the hospital.

#### 3.3.9 Patients 9 (UPN 9909)

A 50‐year‐old man with diabetes mellitus and hyperlipoproteinaemia was admitted with myalgia, cough and dyspnoea. Oxygen support was initiated immediately (10 L/min), and the oxygen saturation level at admission was 88% and 74% with and without a face mask respectively. The patient was restless and unwilling to cooperate with the medical staff. His condition worsened, and 4 days after admission and despite supportive care, his oxygen levels dropped to 89% and 65% with and without a mask respectively. To improve oxygenation, the face mask was upgraded to a reservoir bag. A CT scan indicated bilateral and multifocal ground‐glass opacities. Five days after admission and despite medical treatment, the respiratory distress worsened, and the patient became unstable. Consequently, DSCs were infused. Oxygen saturation stabilized between 89% and 92%. To improve oxygenation, BiPAP was applied. Three days later, a second dose of DSCs was infused. Subsequently, the patient's general condition stabilized, and oxygen saturation levels reached 80%–88%. The patient was agitated, irritable and insisted on being discharged. Psychological counselling was ineffective, and finally, he left the hospital despite the high mortality risk. He refused all medical care, and 2 days later, he died at home due to cardiac arrest.

#### 3.3.10 Patients 10 (UPN 9910)

A 63‐year‐old woman with a history of breast cancer and mastectomy was admitted with dyspnoea and fever. On admission, the oxygen saturation level was 78% (without a mask) and oxygen 10 L/min was started. Lung CT scan showed multifocal infiltration of both lungs. Oxygen saturation decreased, and after 3 days, the face mask was upgraded to a reservoir bag supplying 12 L/min oxygen. Blood oxygen levels barely reached 69%. After 4 days of conventional treatment, a CT scan showed that the opacities in the lungs had increased, and the patient's condition worsened. Five days after admission, one dose of DSCs was infused. Twenty four hours later, oxygen saturation levels reached 84% (without a mask), the patient's condition stabilized, and disease progression stopped. Three days after the DSC therapy, oxygen saturation levels stabilized above 88% (without a mask), and a control CT scan indicated decreased opacities of lung infiltration. Accordingly, oxygen supply was reduced to 5 L/min and the face mask changed to a nasal tube. Oxygen without a mask reached 89%. Eight days after a single DSC infusion, the patient was discharged from the hospital in good health and no need for oxygen.

### 3.4 The effect of DSC therapy on inflammatory cytokines

**FIGURE 3:**
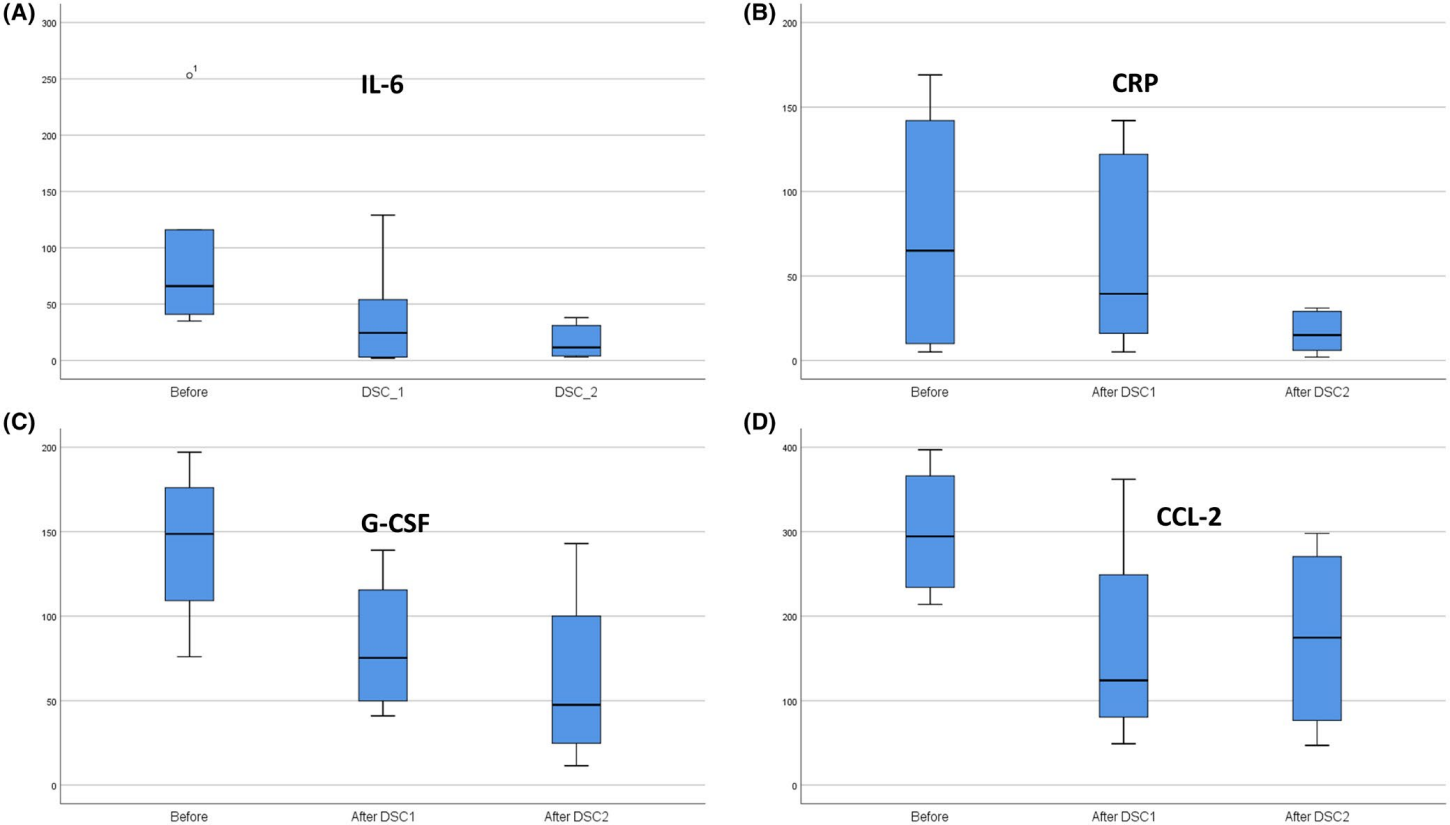
Changes in the levels of cytokines and inflammatory mediators following decidua stromal cells (DSC) treatment. (A) Median levels of IL‐6 before and after the first and second DSC infusions (PV = 0.028, *N* = 7). (B) Median levels of CRP before and after the first and second DSC infusions (PV = 0.028, *N* = 7). (C) Median levels of G‐CSF before and after the first and second DSC infusions (*N* = 7). (D) Median levels of CCL‐2 before and after the first and second DSC infusions (*N* = 7). In the graphs, ‘base’ means 1 day before DSC therapy; ‘after DSC 1’ means 1 day after the first DSC infusion, and ‘after DSC 2’ means 1 day after the last DSC infusion or on the day of discharge

The acute inflammatory mediators, *for example* IL‐6, CRP, CCL‐2 and G‐CSF, were measured at different time points before and after DSC therapy in 7 of the 10 patients. The median level of IL‐6 before DSC therapy was 69.3 pg/ml, which decreased to 11 pg/ml after two DSC infusions (*p* < 0.028) (Figure 3A). Plasma levels of CRP before and after DSC therapy were 57 and 13.5 mg/L respectively (*p* < 0.028) (Figure 3B). Similar reductions were observed in the serum levels of GCSF and CCL2; the levels of these cytokines decreased in five out of seven patients (Figure 3C,D).

### 3.5 The effect of DSC therapy on oxygen saturation

**FIGURE 4:**
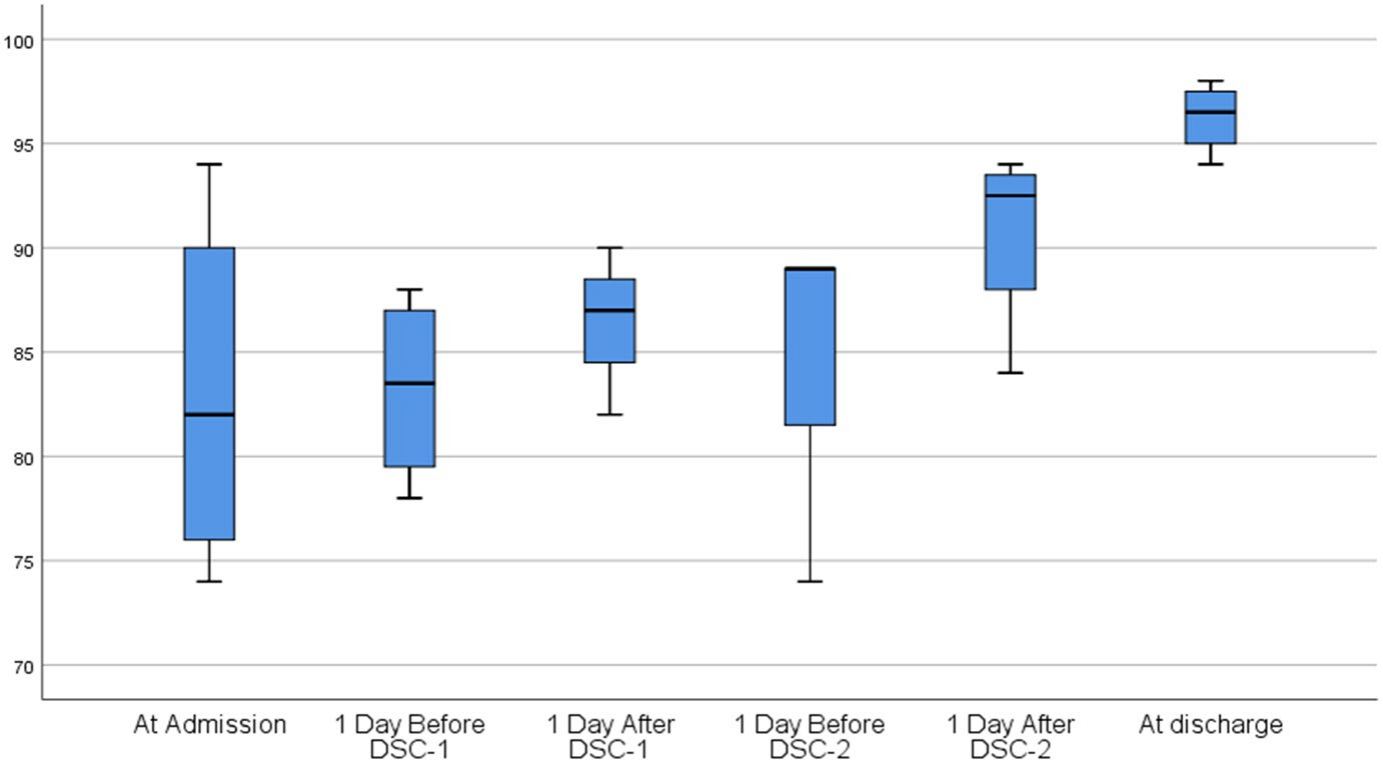
Changes in median blood oxygen levels in patients (*N* = 8) at admission, before and after each decidua stromal cells (DSCs) infusion, and at the time of discharge

We noticed an immediate and durable effect of the DSC infusions on blood oxygenation in all patients (Figure 4, Table 3). The median blood oxygen saturation level before DSC therapy was 80%; after DSC, treatment increased to 95% (Table 3, *p* < 0.012). The DSC infusions had a rapid effect on blood oxygen levels (Figure 4); Twenty four hours after each DSC infusion, oxygen saturation levels continued to increase, and finally, after the full DSC regimen, reached normal levels of >95% in all patients (*p* < 0.028).

### 3.6 Side effects of DSC therapy

Short‐ and long‐term side effects related to the cell infusions were evaluated in all patients. A transient elevation in systolic blood pressure was noted in two patients. This was resolved without any medication or discontinuation of the cell infusion therapy. We did not observe any DSC infusion‐related toxicity or side effects.

## 4 DISCUSSION

The cytokine storm and subsequent ARDS are unpleasant complication of COVID‐19 infection.^6^, ^7^, ^9^ ARDS develops in 42% of patients with COVID‐19 pneumonia, and 61%–81% of these patients require intensive care.^23^ To decrease mortality rate, the therapeutic strategy should control the cytokine storm and prevent ARDS.^24^ We have previously shown that DSCs have a strong immunomodulatory function and can successfully control steroid‐refractory acute GVHD which is triggered by a cytokine storm.^25^

In the present study, we observed a dramatic decrease in cytokine levels following infusion of DSCs. Levels of IL‐6, G‐SF, CRP and CCL2 all decreased. Inflammatory mediators play a pivotal role in the initiation and promotion of ARDS following COVID‐19 infection.^26^ Despite the important role of IL‐6 in initiation and promotion of the cytokine storm, DSC therapy is certainly more effective for controlling the cytokine storm than anti‐IL‐6 monoclonal antibodies. In a randomized, double‐blind, placebo‐controlled trial, the anti‐IL‐6 antibody tocilizumab was given to 141 patients with COVID‐19‐induced ARDS. The results of this study indicated that tocilizumab was not effective and was also associated with a higher rate of adverse effects compared with the control group.^27^

Oxygen levels in the blood are important prognostic and predictive factors for the outcome of COVID‐19‐induced ARDS.^28^ Xie et al.^28^ showed that hypoxaemia was independently associated with in‐hospital mortality. High SpO_2_ levels after oxygen supplementation were associated with reduced mortality which was independent of age and gender.^28^ In the present study, we saw increased PO_2_ levels both as a rapid response following DSC infusion and as durable improvement in blood oxygen levels. Nine out of ten treated patients felt less chest tightness and less dyspnoea after DSC infusion. We have previously reported a similar dramatic effect of DSCs in ALI/ARDS induced by septicaemia caused by alpha‐haemolytic streptococci in a neutropenic patient.^18^

Lung involvement is seen in a majority of patients with COVID‐19‐induced ARDS, mainly as ground‐glass opacity or consolidation on a plain chest X‐ray or CT scan.^29^ It was proposed that changes on CT can predict the outcome of the disease.^29^ We could see a rapid reversal of ground‐glass opacity or consolidation on CT images following DSC treatment. Liu et al reported disappearance of pulmonary sequelae up to 3 weeks after discharge in more than 50% of the patients.^30^ Interestingly, in our study, we observed that signs of lung pathology on CT scans disappeared within 10 days in all patients, which is faster than in Liu's report. This may be due to the strong anti‐inflammatory effect of DSCs.

In the present study, three patients received one dose of DSCs, six patients received two doses, and one patient received three doses. The number of doses required to achieve a response depends on the severity of the disease. When DSCs were used to treat acute GVHD, the number of doses needed to reverse the disease also varied. Some patients responded to a single dose, whereas some patients needed two or more doses of DSCs.^13^ In the present study, the response of the ARDS symptoms also differed between the patients. Some patients had an early response following a single dose of DSCs, while others required additional doses before complete symptom resolution was achieved. This may reflect the severity and magnitude of the cytokine storm in COVID‐19‐induced ARDS in different patients.

It may be debated which type of MSCs should be used to treat clinical ARDS. A randomized study was performed using BM‐MSCs at a dose of 10 × 10^6^ cells/kg.^17^ BM‐MSC therapy was not associated with better outcomes compared with the placebo group. In the above‐mentioned report,^17^ cell viability was 36%–85%, compared with more than 95% for the DSCs used in our study. The ten times higher cell dose could probably not compensate for the poor cell survival. There are several other cell‐based therapies for COVID‐19‐induced ARDS patients reported.^11^, ^12^ Meng et al.^31^ reported a randomized trial using umbilical cord blood MSC (UC‐MSC) which demonstrated a favourable safety profile for UC‐MSC; however, the efficacy of UC‐MSC therapy was not superior to the control group. The mean levels of CRP before cell therapy were lower in this study (Median of 2.98 [1.2–36.4]) than what we observed in our patients (Median of 57 [7.1–169]). This may reflect that our patients had a more severe cytokine storm compared with those in the report by Meng et al. Adipose‐derived MSCs failed to show efficacy in a small, randomized study in ARDS.^19^ Compared to MSCs from BM, UC or fat, DSCs may seem preferable for the treatment of ARDS.

It has been debated if MSCs should be fresh or frozen.^32^, ^33^ We have thawed frozen cells in this and all our clinical studies and experimental studies.^13^, ^15^, ^16^, ^18^ Most importantly, cell viability was always above 90% for the DSCs given to the patients in the present study. This compared favourably with another study using MSCs for ARDS.^17^ The outcome in the present study and in others using DSCs emphasizes that mesenchymal stromal cell viability is probably more important than if the cells were used fresh or thawed from frozen cells.^13^, ^15^, ^17^, ^34^

One of the most important aims in a pilot study of a new treatment modality is evaluating the side effects and any SAEs. Two patients died during the treatment, and one patient died because he deliberately refused all therapy and left the hospital. The first patient died due to cardiac arrest, and MOF was the cause of death in the second patient. The cardiac arrest occurred in a patient with known IHD who was affected by COVID‐19‐induced ALI/ARDS. Despite an increase in PO_2_ and significantly decreased levels of IL‐6, CRP and GCSF, which demonstrated the efficacy of the DSC therapy, the patients died 3 days later. The patient who developed MOF had a history of epilepsy and convulsions. It is possible that the corona virus infection may have infected several other organs apart from the lungs, including the CNS, which may have led to MOF and death. This occurred despite decreased levels of almost all inflammatory mediators in this patient.

None of the patients experienced an SAE that was related to the DSCs infusions. Increased systolic blood pressure was observed in two patients when they received DSCs; however, blood pressure control was achieved without any medication or discontinuation of the DSC infusions. This is consistent with the previous experience from using DSCs.^15^ The safety of DSCs is also well established from animal data.^16^ Furthermore, a clinical toxicity study in patients treated for acute GVHD and haemorrhagic cystitis showed only three minor transfusion reactions in 40 patients treated with DSCs.^15^ MSCs and DSCs may not have completely comparable toxicity as there are some obvious differences. For example, DSCs have a stronger haemostatic procoagulant effects than BM‐MSCs, which is due to a higher expression of procoagulant tissue factor being 39% compared to 6% for BM‐MSCs.^35^ Pre‐coagulant tissue factor (CD142) expression may be important when treating critically ill COVID‐19 patients with an underlying coagulopathy.^36^ COVID‐19 patients have an increased risk of thromboembolism.^37^ The patients were flushed with 5000 IU of heparin intravenously before and after DSCs infusion. This may be important for safe infusion in COVID‐19 patients.^38^ However, none of the patients in this study developed thromboembolism following infusion of DSCs. This is consistent with the experience to date from using DSCs for acute GVHD and haemorrhagic cystitis.^13^, ^15^ From this study, it seems that DSCs are also safe for administration to COVID‐19‐infected patients with pulmonary disease.

Corticosteroid is a useful treatment for COVID‐19 ARDS patients. However, it is not curative.^39^ Chaudhuri et al.,^39^ in a comprehensive review paper, showed that corticosteroid use may reduce ICU mortality (RR 0.61, 95% CI 0.38–0.99) and probably reduce hospital mortality (RR 0.67, 95% CI 0.46–0.96) in critically ill patients with ARDS. In line with this observation, some of our patients received concomitant corticosteroid therapy. To explore and exclude confounding effects of corticosteroid administration, controlled randomized studies are needed.

Among the COVID‐19 ALI/ARDS patients, DSC gave no toxicity or infusion‐related adverse effects. DSC therapy decreased the levels of cytokines IL‐6, G‐CSF, CRP and CCL 2. There was an increase in oxygenation and reversal of pulmonary disease. Four patients could be discharged after a few days. Two patients with COVID‐19‐induced ARDS and additional serious medical problems died of cardiac arrest and MOF respectively. We conclude that DSC therapy could reverse the cytokine storm and should preferably be given to patients with COVID‐19 disease with ARDS. Further studies including controlled randomized trials are required.

## CONFLICT OF INTEREST

Dr Behnam Sadeghi is founder of Sibcell Biotech. All other authors declare no competing interests.

## AUTHOR CONTRIBUTIONS

**Behnam Sadeghi:** Conceptualization (lead); Data curation (equal); Funding acquisition (lead); Investigation (lead); Methodology (equal); Project administration (lead); Resources (lead); Supervision (lead); Validation (equal); Writing‐original draft (lead); Writing‐review & editing (lead). **Elham Roshandel:** Conceptualization (equal); Data curation (equal); Formal analysis (equal); Investigation (equal); Methodology (equal); Project administration (equal); Visualization (equal); Writing‐original draft (equal). **Ali Pirsalehi:** Conceptualization (equal); Data curation (equal); Funding acquisition (equal); Investigation (equal); Methodology (equal); Project administration (equal); Supervision (equal); Validation (equal); Writing‐original draft (equal). **Sepide Kazemi:** Conceptualization (equal); Data curation (equal); Formal analysis (equal); Investigation (equal); Methodology (equal); Project administration (equal); Validation (equal); Writing‐original draft (equal). **Ghazaleh Sankanian:** Data curation (equal); Formal analysis (equal); Investigation (equal); Methodology (equal); Writing‐original draft (equal). **Mohammad Majidi:** Conceptualization (equal); Data curation (equal); Formal analysis (equal); Investigation (equal); Methodology (equal); Software (equal); Writing‐original draft (equal). **Maryam Salimi:** Conceptualization (equal); Data curation (equal); Investigation (equal); Methodology (equal); Writing‐original draft (equal). **Nasser Aghdami:** Conceptualization (equal); Data curation (equal); Formal analysis (equal); Investigation (equal); Methodology (equal); Supervision (equal); Writing‐original draft (equal). **Hoda Sadrosadat:** Conceptualization (equal); Data curation (equal); Investigation (equal); Methodology (equal); Writing‐original draft (equal). **Sarvenaz Samadi Kochaksaraei:** Conceptualization (equal); Data curation (equal); Investigation (equal); Methodology (equal); Writing‐original draft (equal). **Farshid Alaeddini:** Conceptualization (equal); Data curation (equal); Formal analysis (lead); Investigation (equal); Methodology (equal); Writing‐original draft (equal). **Olle Ringdén:** Conceptualization (equal); Formal analysis (supporting); Funding acquisition (supporting); Investigation (supporting); Methodology (supporting); Resources (supporting); Writing‐original draft (lead); Writing‐review & editing (equal). **Abbas Hajfathali:** Conceptualization (equal); Data curation (equal); Investigation (supporting); Methodology (supporting); Project administration (supporting); Supervision (supporting); Writing‐original draft (equal); Writing‐review & editing (equal).

## Supporting information

Supplementary Files

## Data Availability

Data available on request due to privacy/ethical restrictions.
